# Phenotypic, functional, and metabolic heterogeneity of immune cells infiltrating non–small cell lung cancer

**DOI:** 10.3389/fimmu.2022.959114

**Published:** 2022-08-10

**Authors:** Beatrice Aramini, Valentina Masciale, Anna Valeria Samarelli, Alessandra Dubini, Michele Gaudio, Franco Stella, Uliano Morandi, Massimo Dominici, Sara De Biasi, Lara Gibellini, Andrea Cossarizza

**Affiliations:** ^1^ Division of Thoracic Surgery, Department of Experimental, Diagnostic and Specialty Medicine—DIMES of the Alma Mater Studiorum, University of Bologna, G.B. Morgagni—L. Pierantoni Hospital, Forlì, Italy; ^2^ Division of Oncology and Laboratory of Cellular Therapies, Department of Medical and Surgical Sciences, University of Modena and Reggio Emilia, Modena, Italy; ^3^ Division of Pathology, G.B. Morgagni—L. Pierantoni Hospital, Forlì, Italy; ^4^ Division of Thoracic Surgery, Department of Medical and Surgical Sciences, University of Modena and Reggio Emilia, Modena, Italy; ^5^ Department of Medical and Surgical Sciences for Children and Adults, University of Modena and Reggio Emilia, Modena, Italy; ^6^ National Institute for Cardiovascular Research, Bologna, Italy

**Keywords:** NSCLC, tumor infiltrated immune cells, immunometabolism, cancer stem cells, tumor-infiltrating myeloid cells

## Abstract

Lung cancer is the leading cancer in the world, accounting for 1.2 million of new cases annually, being responsible for 17.8% of all cancer deaths. In particular, non–small cell lung cancer (NSCLC) is involved in approximately 85% of all lung cancers with a high lethality probably due to the asymptomatic evolution, leading patients to be diagnosed when the tumor has already spread to other organs. Despite the introduction of new therapies, which have improved the long-term survival of these patients, this disease is still not well cured and under controlled. Over the past two decades, single-cell technologies allowed to deeply profile both the phenotypic and metabolic aspects of the immune cells infiltrating the TME, thus fostering the identification of predictive biomarkers of prognosis and supporting the development of new therapeutic strategies. In this review, we discuss phenotypic and functional characteristics of the main subsets of tumor-infiltrating lymphocytes (TILs) and tumor-infiltrating myeloid cells (TIMs) that contribute to promote or suppress NSCLC development and progression. We also address two emerging aspects of TIL and TIM biology, i.e., their metabolism, which affects their effector functions, proliferation, and differentiation, and their capacity to interact with cancer stem cells.

## Introduction

Lung cancer, both small cell lung cancer (SCLC) and non–small cell lung cancer (NSCLC), is the leading cause of cancer mortality worldwide with 1.8 million deaths per year (1). It is considered the second most common cancer in men, after prostate cancer, and in women, after breast cancer (2), and in 2018, the death for this pathology accounted for the 18.4% of all cancer deaths worldwide (Global Cancer Observatory, https://gco.iarc.fr/today). According to the last GLOBOCAN, 2,094,000 new cases of lung cancer were registered in 2018, and among them, NSCLC is the most prevalent. According to the National Institue of Health (NIH) Surveillance, Epidemiology, and End Results Program, the 5-year survival rate for NSCLC was 26% from 2011 to 2017, which is two-fold higher that in 1975, which was 12% (https://seer.cancer.gov/archive/csr/1975_2016/).

Surgical resection remains the best curative choice in patients with early-stage lung cancer and can also represent an option, together with other treatments, including targeted therapies and immunotherapies, for selected patients with advanced-stage lung cancer. The advent of new therapies and the expansion of treatment options have dramatically improved the clinical outcome of patients with lung cancer (3, 4). However, two main aspects still have a major impact on the clinical management of patients. On the one hand, the recurrence rate after surgery in lung cancer, and in particular in NSCLC, is high, ranging from 20% to 75% of patients in the first 5 years, with most recurrences taking place within the first 2 years after surgery. On the other hand, only a fraction of patients responds to immunotherapies (5). For this reason, during the last years, considerable efforts have been devoted to deconvolute the molecular and cellular mechanisms that support clinical resistance to therapies and/or recurrence after (6).

Extensive evidence suggests that the evolution and characteristics of tumor microenvironment (TME) could play a key role in determining the overall patient propensity to experience recurrence or respond to specific therapeutic intervention (7). TME consists of a heterogenous population of cancer cells together with a variety of infiltrating immune and non-immune host cells, secreted molecules, and extracellular matrix proteins. TME indeed includes T lymphocytes, as well as B lymphocytes, natural killer (NK) cells, dendritic cells (DCs), myeloid-derived suppressor cells (MDSCs), tumor-associated macrophages (TAMs), tumor-associated neutrophils (TANs), cancer-associated fibroblasts (CAFs), adipocytes, vascular endothelial cells, and pericytes (7, 8). Recent data have described the presence of cancer stem cells (CSCs), which exert a pivotal role in the onset, progression, and drug resistance of the tumor itself. As an example, an increase of 1% in CSCs yielded a 26% of an elevated recurrence risk in patients with NSCLC, demonstrating that these cells could be involved in cancer relapse (9). An additional layer of complexity is represented by the fact that a reciprocal communication is present between CSCs and infiltrating immune cells and that this crosstalk simultaneously induces CSCs and tailors the immune response to facilitate tumor immune evasion, metastasis formation, and recurrence (10).

Herein, we present an updated image of the TME in NSLSC, with emphasis on the immune microenvironment and to CSCs that elude the immunologic surveillance. In particular, we discuss the phenotypic, functional, and metabolic characteristics of main subsets of tumor-infiltrating T lymphocytes and tumor-infiltrating myeloid cells (TIMs) and will explore the basis of the interaction(s) between immune cells and CSCs.

## Tumor-infiltrating lymphocytes in NSCLC

### Immunological composition of TME

The immunological analysis of the TME (i.e., the immunoscore) may suggest improved prognosis and predict the response to immunotherapy. Investigations on the cell composition of tumor infiltrating cells reveal that T cells predominate the lung cancer environment (with a mean value of 47% of all immune cells), where CD4^+^ T cells are the most represented T-cell population (26%), followed by CD8^+^ T cells (22%). Here, CD4^−^CD8^−^ T cells represent a small proportion of whole T cells (1.4%). The second most represented cell population is formed by CD19^+^ B cells (16%). Macrophages and NK cells represent 4.7% and 4.5% of the immune cell infiltrate, respectively, whereas DCs are about 2.1%. Regarding granulocytes, neutrophils are 8.6% (being this percentage very variable among patients), mast cells are 1.4%, basophils are 0.4%, and eosinophils are 0.3% (11).

### The role of T cells and their phenotypic and functional heterogeneity

Tumors display different immune infiltration together with different mechanisms of antigen presentation. In particular, TME exerts a strong selection pressure in the early stage that results in ongoing immunoediting in immune-infiltrated tumor regions (12). Not many tumor-infiltrating lymphocytes (TILs) recognize tumor antigens, and little information is available about the mutation-associated neoantigen–specific TIL. The majority of them are cytolytic with transcriptional programs of tissue-resident memory (TRM) cells (13).

TRM cells are lymphocyte residing in the tissues without recirculating, being transcriptionally, phenotypically, and functionally distinct from recirculating central and effector memory T cells. *KLF2* and *S1PR1* are expressed at low level, although they express high levels of CD69. The combination of these molecules avoids exit from the tissue. Moreover, TRM cells, found in the epithelial barriers, could also express α_e_β_7_ integrin for maintenance (the chain α_e_ is also called CD103) [reviewed in (14)]. CD103 binds to the epithelial cell marker E-cadherin; this binding determines the retention of TRM cells in epithelial tumor islets and the further maturation of cytotoxic immune synapse with specific cancer cells, resulting in T-cell receptor (TCR)–dependent target cell killing. In addition, CD103 integrin triggers two different signals that cooperate with TCR, enabling T-cell migration and optimal cytokine production [Interferon gamma (IFN-γ) and Tumor necrosis factor (TNF)]. Indeed, TRM cells infiltrating human NSCLC tumors also express inhibitory receptors, such as Programmed death-1 (PD1), and the neutralization with anti-PD1 enhances CD103-dependent TCR-mediated cytotoxicity toward autologous cancer cells. For this reason, accumulation of TRM cells at the tumor site explains the more favorable clinical outcome and might be associated with the success of immune checkpoint blockade (ICB) in a fraction of cancer patients (15–19).

Two immunophenotypes of TRM could be predictive of clinical outcome: one is activated, expressing Ki67, CD103, PD1, T cell immunoglobulin and mucin domain-containing protein 3 (TIM-3), and Inducible T-cell costimulator (ICOS), and is associated with poor prognosis; and the other is more cytolytic and is associated with good prognosis (20). Moreover, two additional CD8^+^ TIL subpopulations expressing memory-like genes have been reported: one population is represented by circulating precursors and the other is represented by tissue-resident precursors in the juxta-tumor tissue. These two precursor populations become terminally differentiated cells, often dysfunctional or exhausted (21). High ratio of “pre-exhausted” cells, i.e., cells that exhibit hallmarks of both exhausted and memory cells, to exhausted T cells is associated with better prognosis of lung adenocarcinoma (22).

Mucosal associated invariant T (MAIT) cells is a population of CD8^+^ effector memory T cells with pro-inflammatory, cytotoxic, and homing properties. Usually, this population exerts its function in the mucosae where it patrols and orchestrates the immune response. MAIT cells respond to microbial proteins presented by non-polymorphic MHC class I–related molecule (MR1). Recently, it has emerged that a population of MAIT cells, named MR1T cells, can recognize and kill a diverse range of MR1-expressing tumor cells [reviewed in (23)]. MAIT cells are also present in TME of patients with melanoma, but their role is still under investigation (24, 25). As an example, recent data obtained in mice model of lung cancer show that MAIT cells promote tumor initiation, growth, and metastasis. MR1-expressing tumor cells activate MAIT cells to reduce NK-cell effector function, partly in a manner depending on the production of IL-17A (26).

During several chronic infections and cancer, T cell could become exhausted or dysfunctional. Exhaustion is characterized by scarce effector function, well evidenced by the expression of inhibitory receptors and a transcriptional state distinct from that of functional effector or memory T cells. Exhaustion impedes an optimal control of infection and tumors (27). The most common molecules of exhaustion expressed by CD8^+^ TILs are PD1, LAG3, TIM3, TIGIT, CD244, and CD160, along with CD39, which is an ectonucleotidase first identified as an activation marker on human lymphocytes (28) and then as a hallmark of regulatory T cells (Tregs) (29). CD39 hydrolyzes extracellular ATP and ADP into AMP, which is then processed into adenosine by CD73 (30). Adenosine binds to A2A receptors expressed by lymphocytes inducing accumulation of intracellular cAMP, inhibiting T-cell activation and NK cytotoxicity (31). Terminally exhausted CD8^+^ T cells express CD39 (32), and CD39^−^CD8^+^ TILs define populations that lack hallmarks of chronic antigen stimulation at the tumor site. Furthermore, CD39 expression among CD8^+^ TILs correlated with several important clinical parameters such as the mutation status of lung tumor epidermal growth factor receptors (33, 34).

### Lymphocyte infiltration and TLS formation: The role of B and Tfh cells

In addition to the expression of markers of tissue residency and exhaustion, the expression of migratory receptors together with their ligands is dysregulated in TIL subpopulations. CXCR6 is a molecule that characterizes dysfunctional T cells in cancer. The migratory axis CXCR6/CCL16 regulates the localization of TRM to the lung. In a mouse models, CXCR6 expression increased after checkpoint blockade, and it was diminished by TCF1 (35). Moreover, CD8^+^ T cells characterized by high expression of PD1 show a markedly different transcriptional and metabolic profile, showing an impaired production of classical effector cytokines and of CXC-chemokine ligand 13 (CXCL13), which mediates immune cell recruitment to tertiary lymphoid structures (TLSs) (35). CXCL13 is a chemoattractant for B cells, suggesting that B cells may be involved in the formation of TLSs (36).

TLSs are somehow similar to lymph nodes, as they include structures that resemble the germinal centers with follicular DCs and proliferating B cells. T cells surround the germinal centers, together with DCs, plasma cells, lymphatics, and blood vessels [reviewed in (37–39)]. TLSs have been found in different types of human cancers and usually have positive prognostic value in non–small cell lung carcinoma (20, 40, 41). In particular, positive prognostic markers are B cells infiltrating the stroma, the immune response mediated by B cells and the high density of TLS germinal center B cells (42–45). B cells that infiltrate the tumor could display both pro-tumor and anti-tumor activities on the basis of the composition of the TME, on the phenotypes of B cells, and the antibodies production (45). Indeed, two major subtypes of tumor-infiltrated B cells coexist, namely, the naiüve-like and plasma cell–like B cells. The naiüve-like B cells are decreased in advanced NSCLC, and their lower level is associated with poor prognosis as they suppress the growth of tumor cells. Plasma cell–like B cells counteract cancer cell growth in the early stage of NSCLC, but they can favor cell growth in the advanced stage of NSCLC (46). Moreover, high B-cell density within TLSs can counterbalance the damaging effect of high Treg density on patient survival (36).

Follicular helper T (Tfh) cells have been found also in the TLS, cooperating with B cells (47). Tfh cells are memory CD4^+^ T cells expressing PD1, ICOS, and CXCR5 [reviewed in (48)]; they play a pivotal role in orchestrating the humor immune response contributing to antibody affinity maturation following B-cell isotype switching in the germinal center. TLS formation is strongly dependent on CXCL13 signaling, potentially due to its effect on B-cell recruitment by Tfh cells, whose percentage is increased in the tumor due to high concentration of transforming growth factor–β (TGF-β that promotes their differentiation (49–51).

### The role of regulatory T cells

Further heterogeneity has been reported within Tregs present in the tumor, which is characterized by the expression of activation molecules such as TNF receptor superfamily member 9 (TNFRSF9) and Interleukin 1 receptor type 2 (IL1R2). This population correlates with poor prognosis in lung adenocarcinoma (22). Tumors are enriched by Tregs, where they dampen the anti-tumor immune response (52). Tregs are critical in self-tolerance and immune homeostasis. They express the transcription factor forkhead box P3, a master regulator of Treg differentiation and suppressor function. Tregs exert their suppressive function by different mechanisms: 1) producing and releasing immunosuppressive cytokines (IL-10 and TGF-β); 2) constitutively expressing high levels of CD25 that binds and utilizes IL-2; and 3) expressing inhibitory molecules such as Cytotoxic T-lymphocyte antigen 4 (CTLA-4), PD1, and LAG3 (53).

In TME, Tregs (tumor-infiltrating Tregs, or ti-Tregs) induce the suppression of anti-tumor immune responses together with the development of an immunosuppressive TME. Tregs are recruited in TME by chemotaxis as tumor cells and tumor-associated cells secrete chemokines that attract Tregs. These ti-Tregs inhibit the activity of effector T cells and facilitate tumor growth. Chemokines and chemokine-receptor axis that are majorly involved in this chemotaxis of Tregs are CCL28-CCR10, CCL5-CCR5, CCL22-CCR4, and CXCL9/10/11-CXCR3 (54). Ti-Treg cells are highly suppressive, upregulate several immune-checkpoints, and express on the cell surface molecules such as IL1R2, PDL1, PDL2, and CCR8. In these cells, high expression of genes such as *LAYN*, *MAGEH1*, or *CCR8* correlates with poor prognosis (55, 56). Moreover, *IRF4* Tregs express suppressive molecules, and in NSCLC, their presence correlated with multiple exhausted subpopulations of T cells. *IRF4* either alone or in combination with its partner *BATF* directly controls a molecular program responsible for immunosuppression in tumors. The abundance of *IRF4* Tregs correlates with poor prognosis (57).

### The role of NK cells

**Figure 1:**
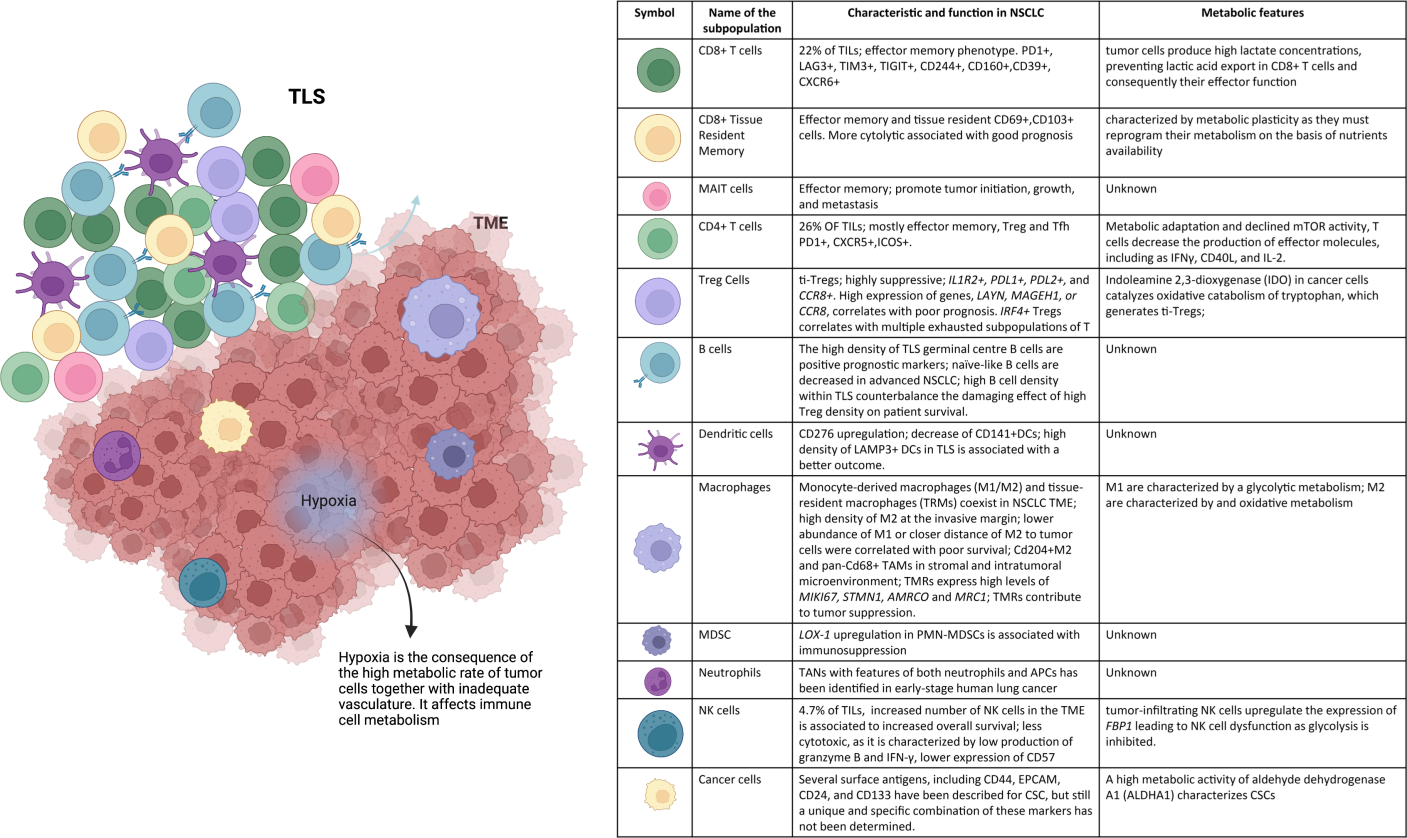
NSCLC microenvironment. The tumor niche is a dynamic structure in which tumor cells coexist with tumor vasculature, extracelluar matrix, and immune cells. The immune infiltrate includes multiple cell types, CD8^+^ T cells, CD4^+^ T cells, mucosal-associated invariant T (MAIT) cells, regulatory T (Treg) cells, B cells, NK cells, DCs, macrophages, MDSCs, and neutrophils. These cell subsets can have both pro- and anti-tumor functions and can vary in their activation status, their metabolism, and their localization within the tumor. Hypoxia drives changes in immune cell metabolism and functions. TLSs within the TME consist of T-cell areas containing DCs and B-cell areas with germinal centers. They represent critical sites where specific T and B cells can undergo terminal differentiation into effector cells.

NK cells, effector cells among the innate lymphoid cells (ILCs), have been isolated from TME, even at low percentage. These cells express a variety of activating and inhibitory receptors, together with chemokine receptors and regulatory/cytotoxic molecules such as CD16 (FcγRIII receptor) and CD56 (neural cell adhesion molecule). Historically, different populations of NK cells can be identified on the basis of the expression of CD16, CD57, and CD56 (58, 59). In the periphery, highly cytotoxic NK cells are CD56^+/−^CD16^++^, while those immunomodulatory and able to produce cytokines, residing in the lymph nodes, are CD56^++^CD16^−^ [reviewed in (60)]. NK cells are found at low frequency within tumors (61), and an increased number of such cells in the TME are associated to an increased overall survival (OS) (62). NK cells reside in the tumor stroma, not directly in contact with cancer cells, and have a phenotype similar to that of circulating CD56^++^ NK cells (61). However, tumor-resident NK cells are less cytotoxic, being characterized by a low production of granzyme B and IFN-γ and a low expression of CD57 (63). The possible explanation is that TME negatively regulates the maturation, maturation, proliferation, and effector function of NK cells. Locally produced TGF-β can skew NK cells toward ILC type 1 (ILC1), which are not cytotoxic. TGF-β is also able to diminish recruitment of CD56^+/−^ cells and favors that of CD56^++^ (64). The main phenotypic, functional, and metabolic characteristics of different subpopulation described in this paragraph are reported in **Figure 1**.

### TILs metabolism

**Figure 2:**
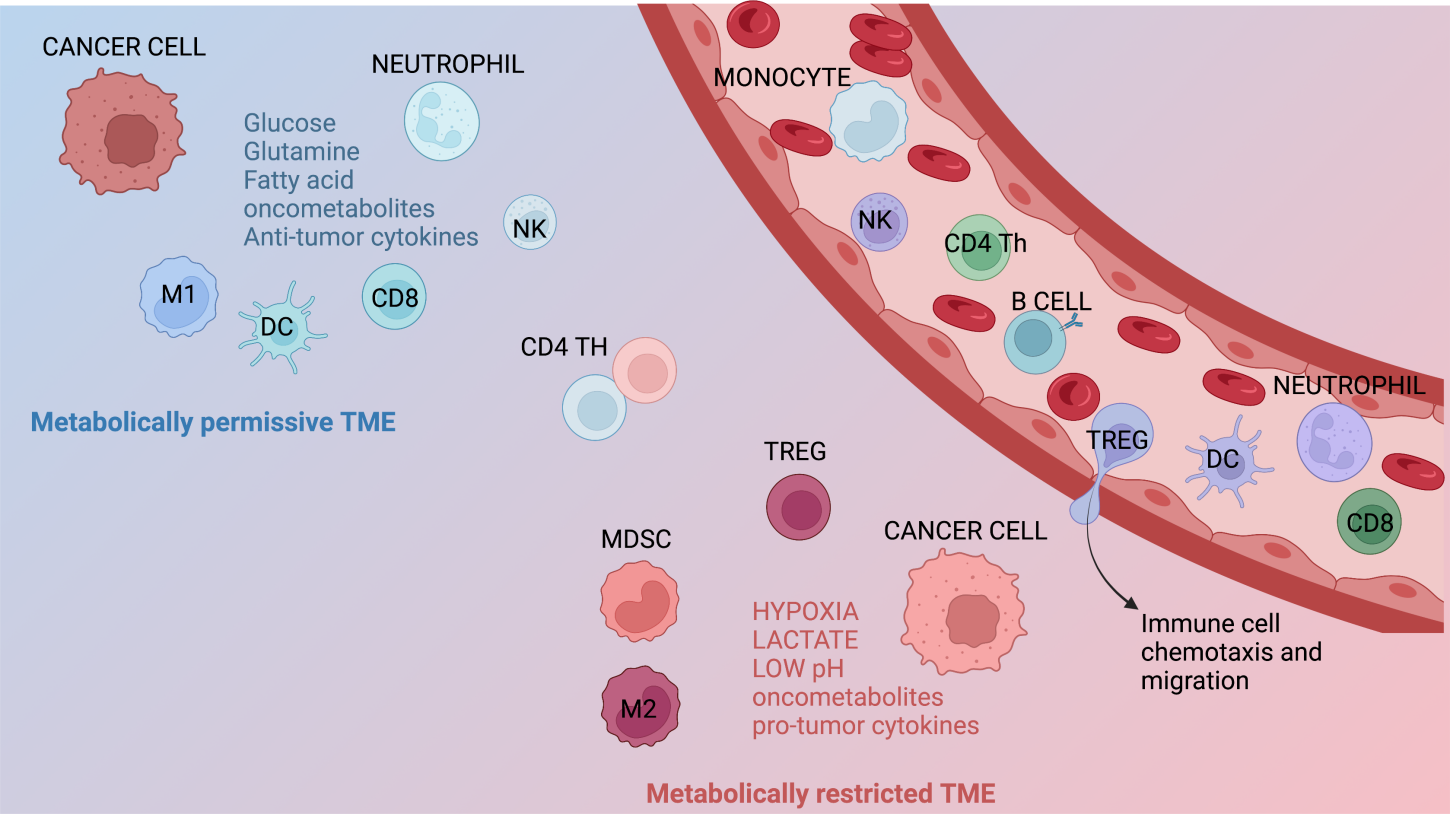
Metabolic features of the TME. Immune infiltrates contribute to generate metabolically permissive or restricted tumor microenvironment (TME). Immune populations that generally lead to a metabolically permissive TME are neutrophils, M1 macrophages, dendritic cells (DCs), NK cells, and CD8^+^ T cells. Immune subsets generating metabolically restricted TME are represented by M2 macrophages, regulatory T (Treg) cells, and myeloid-derived suppressor cells (MDSCs). Several other factors, including hypoxia, restricted/augmented nutrient availability, low pH, the presence of metabolites/oncometabolites, and/or specific cytokines or chemokines, can also contribute affect the metabolic characteristics of the TME.

Lymphocytes must adapt to a wide array of environmental stressors during their physiological development, when they undergo a profound metabolic remodeling process [reviewed in (65)]. Glycolysis not only provides precursors of biomass and ATP but also allows activated T cells to sustain effector functions through both transcriptional and translational regulations (66). However, T cells are able to survive in glucose-depleted conditions using mitochondrial oxidative phosphorylation (OXPHOS) to support their energy demand (67). In TME, T cells encounter a hostile metabolic environment, as the increased metabolic activities of cancer cells could lead not only to a hypoxic environment but also to the depletion of key nutrients required by TILs. In the TME, immune cells are characterized by metabolic plasticity, being able to reprogram their metabolism on the basis of nutrients’ availability. Glucose, glutamine, and long-chain and short-chain fatty acids can be used by memory T cells to fuel OXPHOS; nevertheless, different memory subsets preferentially use different substrates (**Figure 2**). TRM cells are able to uptake high amounts of fatty acids directly from the microenvironment. Survival of TRM T cells requires exogenous lipid uptake, and indeed: i) FABP4/5 (lipid chaperone) and CD36 are specifically upregulated in TRM cells; and ii) *ex vivo* exogenous supplementation of fatty acids increased spare respiratory capacity (68, 69). As a consequence of metabolic adaptation and declined activity of the mammalian target of rapamycin (mTOR), T cells decrease the production of effector molecules, including IFN-γ, CD154, and IL-2. Exhausted T cells exhibit suppressed mitochondrial respiration and/or glycolysis and such poor metabolic fitness may reinforce T-cell exhaustion. PD-1 regulates early glycolytic and mitochondrial alterations and repressed transcriptional coactivator PGC-1α (70–73).

TME is characterized by hypoxia, which is the consequence of the high metabolic rate of tumor cells together with inadequate vasculature. Hypoxia can have an immunostimulatory or immunosuppressive effect on T cells in the TME [reviewed in (74)]. Hypoxia-inducible factor (HIF-1α) is an oxygen-dependent transcriptional activator, and it exerts a crucial role in the angiogenesis of tumors. The activation of HIF-1α is related to a variety of tumors. Hence, blocking this pathway could inhibit tumor growth and considered to be a target for anticancer therapies (75, 76). Moreover, HIF-1α inhibition together with anti–PD-1 therapy could impair tumor growth *in vitro* and *in vivo*. Public datasets were utilized to investigate patients’ prognosis based on expressions of HIF-1α and CD8^+^ TILs (77). Patients with high HIF-1α expression exhibited low TILs, indicating an immunosuppressive phenotype, whereas HIF-1α inhibition suppressed alleviated tumor immunosuppression.

Finally, nutrient competition and metabolic by-products by tumor cells are deleterious to T cells. In cancer cells, indoleamine 2,3-dioxygenase (IDO) catalyzes oxidative catabolism of tryptophan, diminishing anti-tumor immune responses by sequestrating the essential amino acid and producing kyneurenine, which generates ti-Tregs (78, 79). Moreover, tumor cells produce high lactate concentrations, preventing lactic acid export in CD8^+^ T cells and consequently their effector function (80).

Metabolites serve as messengers to govern cell effector functions, also in the case of NK cells as higher glycolysis and OXPHOS have a functional impact on CD56^++^ NK cells, which are induced to produce IFN-γ (81, 82). On the contrary, the stimulation with IL-2/IL-12 force NK cells to select glycolysis as their main metabolic pathway. In general, activated NK cells are highly glycolytic, and tumor-infiltrating NK cells upregulate the expression of the gluconeogenesis enzyme fructose bisphosphatase 1 (FBP1) through a mechanism the involve TGF-β, leading to NK-cell dysfunction as glycolysis is inhibited. The use of FBP1 inhibitor drifts back the dysfunctional phenotype of NK cells during tumor promotion but not during tumor progression (83).

## Tumor-infiltrating myeloid cells in NSCLC

A number of evidence suggest that myeloid cells of monocytic and granulocytic lineages are also abundant within the lung TME and account for almost 50% of tumor-infiltrating CD45^+^ cells (84). They contribute to carcinogenesis and can also modulate adaptive immunity by controlling TIL composition, activation, and function. TIMs mainly comprise DCs, MDSCs, monocytes, macrophages, and polymorphonuclear granulocytes, mainly neutrophils.

### Myeloid dendritic cells

Myeloid DCs have a central role in anti-tumor immunity for their ability to prime T cells in lymph nodes. CD103^+^ DCs control CD8^+^ T-cell activation, across multiple tumor types (85). However, tumors can inhibit DC function or shape the TME to recruit immune-suppressive DCs. In NSCLC, DCs upregulate B7-H3 (CD276), which is a co-inhibitory receptor, thus failing to stimulate T lymphocytes (86). DCs can also produce TGF-β, which induces differentiation of CD4^+^ T lymphocytes into Tregs, which suppress T-cell proliferation (87). In early-stage lung adenocarcinoma, CD141^+^ DCs, which interact preferentially with CD8^+^ T cells, are profoundly reduced if compared with the normal lung. Conversely, CD1c^+^ DCs, which express IL-6, IL-8, and IL-1 β, a typical cytokine profile of CD14^+^ monocytes, were increased in tumors rather than in the normal lung (63). Again, a high density of lysosomal membrane–associated protein 3–positive DCs in TLSs was associated with cytotoxic T-cell infiltration and better outcome prediction (88, 89). Better prognosis has also been described for patients with NSCLC with elevated calreticulin (CRT) expression on tumor cells that were, in turn, associated with a higher density of infiltrating mature DCs and effector memory T-cell subsets, thus suggesting that, in the TME, CRT triggers the activation of adaptive immune responses (90, 91).

### Myeloid-derived suppressor cells

MDSCs are a heterogenous cell population, generated in several pathological settings, including cancer; derive from a pathologic, non-conventional activation of monocytes and immature neutrophils; and are able to restrain T-cell proliferation and cytokine production (92). Phenotypically, they can be divided into two large subsets, named monocytic (M)– and polymorphonuclear (PMN)–MDSCs. In the vast majority of the studies reported so far, PMN-MDSCs are more frequent than M-MDSCs (93). Although PMN-MDSCs are phenotypically similar to neutrophils, as they share the CD11b^+^CD14^−^CD15^+^/CD66b^+^ phenotype, they have a distinct gene expression profile (94). The greatest differences are represented by the expression of genes associated with endoplasmic reticulum stress. Among these genes, lectin-type oxidized LDL receptor-1 (LOX-1) was upregulated in PMN-MDSCs if compared with total neutrophils (94). The fact that this was associated to a potent immunosuppressive activity and that LOX-1 inhibition can block the suppressive activity of LOX-1 PMN-MDSCs could have a potential therapeutic application in NSCLC (94).

### Monocytes and macrophages

Monocytes play a central role in antigen sensing and presentation, phagocytosis, and cytokine production (95). In terms of metabolic features and functional plasticity, they are heterogeneous, and their plasticity depends on their metabolism (96). Monocytes can infiltrate tumors and can be present in the tumor or can originate from TAMs, which are also part of NSCLC immune infiltrate (88, 97). TAMs are highly plastic cells too and, depending on the local TME, tumor stage, and other factors, exhibit a number of phenotypes, which include, among others, pro-inflammatory (M1) macrophages with anti-tumor activity and anti-inflammatory/immunosuppressive (M2) macrophages with pro-tumor activity. During the last years, the integration of new single-cell technologies, including cytometry by time-of-flight mass spectrometry (CyTOF), assay for transposase-accessible chromatin sequencing, and massive parallel single-cell RNA sequencing, allowed the exploration of macrophage heterogeneity at the highest resolution to move forward the oversimplified dichotomy M1/M2 (98). Given this bursting heterogeneity of phenotypes, understanding the prognostic relevance of TAMs in NSCLC remains complicated, and further studies are needed.

In addition to phenotypic diversity, spatial density and distribution of TAMs may be relevant to tumor progression, as neighboring immune and tumor cells generate complex dynamic interactions that can eventually influence prognosis and response to treatment (8, 99). In NSCLC, a marked heterogeneity of TAMs between invasive margin and tumor center is present, with high M2 density in particular at the invasive margin. Lower abundance of M1 or closer distance of M2 to tumor cells was correlated with poor survival (100). Recently, tissue-resident macrophages (TRMs), which express canonical macrophage genes, including mannose receptor C-type 1 (MRC1), C1QA, CD68, and apolipoprotein (APOE), but lack a monocytic signature, have been described in early-stage, treatment-naïve NSCLC (101). TRMs also express high levels of cell cycle genes MKI67 and STMN1, indicating self-renewal potential, as well as Macrophage receptor with collagenous structure (MARCO) and MRC1. This and other findings indicate that TRMs are self-maintained and do not derive from blood-circulating myeloid progenitors, including monocytes, suggesting that Tissue resident macrophages and monocyte-derived-macrophages likely coexist in the NSCLC TME (101). Tissue resident macrophages contribute to tumor progression, both through direct and indirect mechanisms. They induce epithelial-to-mesenchymal transition (EMT), thus promoting cell invasiveness, and lead to an expansion of Treg cells, thus protecting tumor cells from killing by CD8^+^ T lymphocytes (101). Other reports revealed that MARCO-expressing macrophages blocked cytotoxic T-cell and NK-cell activation, again by enhancing Treg proliferation and by producing IL-10 (102). In addition, previously described TRMs have a distinct temporal and spatial distribution in the TME, as they accumulate close to tumor cells early during tumor formation to promote EMT (101).

Metabolic heterogeneity of TAMs, both derived from monocytes or Tissue resident macrophages, further complicates the attempt to resolve the prognostic relevance of TAMs in NSCLC (103). M1-like macrophages are often associated with highly glycolytic metabolism, whereas M2-like are mainly characterized by an oxidative metabolism. However, this represents another simplified view that does not properly fit with the emerging metabolic heterogeneity of macrophages in TME (104). Main metabolic circuitries emerged that guide the ability of TAMs to influence tumor progression and prognosis. Among these, glucose, glutamine, fatty acid, and oxidative metabolism are main determinants of TAM functions and likely of disease progression. Other metabolic pathways could be relevant but, at present, are completely unexplored. Recently, a role for succinate metabolism has emerged. Cancer cells can release succinate, which is generated from succinyl-CoA by tricarboxylic acid (TCA) cycle enzyme succinyl-CoA synthetase, and that succinate can, in turn, activate succinate receptor (SUCNR1) signaling to polarize macrophages into TAM through the phosphoinositide 3-kinase (PI3K)/HIF-1a axis (105).

### Neutrophils

Neutrophils are numerous in NSCLC specimens and accounting for nearly 20% of all CD45^+^ cells (84). Neutrophils are the most abundant effector cells of the innate immune system, with a primary role in the response against extracellular pathogens and in acute inflammation, and are among the first responders to infection and tissue damage (106). In general, TAN abundance in the TME is considered a leading predictor of a poor outcome (99), and a high intra-tumoral neutrophil-to-CD8^+^ T-cell ratio is a poor prognostic indicator of both recurrence-free-survival and OS (107).

The disease stage as well as the tissue context and the levels and type of inflammatory mediators found in the TME are key determinants of the specific role of these cells in promoting or restraining cancer and may dictate the phenotypes of TANs (108–110). Indeed, TANs can exert dual, clearly opposite, functions in tumor immunity. TANs can indeed take an anti-tumorigenic (N1) phenotype versus a pro-tumorigenic (N2) phenotype. The anti-tumor activities of N1 TANs include expression of immuno-activating cytokines, lower levels of arginase, high H_2_O_2_ production, and increased capability of killing tumor cells *in vitro* (111, 112). N2 phenotype is characterized by higher expression of factors promoting tumor growth, high production of IL-8/CXCL8, and low production of TNF or CXCL10 (111–113). TGF-β and IFN-β are important contributors of TAN polarization (112, 114); although such functional diversity of TANs has been reported mostly in murine tumor models (112), their role in human lung cancer remains elusive. When compared with blood-derived neutrophils, TANs exhibited an activated phenotype (CD62L^low^CD54^hi^); expressed higher levels of several homing receptors, including CCR5, CCR7, CXCR3, and CXCR4; produced increased amounts of pro-inflammatory cytokines, including IL-6 and IL-8; and could stimulate T-cell proliferation and induce IFN-g (115). Recently, a subset of TANs that displayed features of both neutrophils and antigen-presenting cells has been identified in early-stage human lung cancer. These cells originate from CD11b^+^CD15^hi^CD10^−^CD16^low^ immature progenitors and can cross-present antigens and tailor anti-tumor T-cell responses (116).

However, there is little information on how this TAN heterogeneity is established and maintained, and, importantly, studies are scarce on humans as the murine tumor models have been preferentially investigated. Data on the phenotype and function of TANs in patients with lung cancer remain limited and are mostly from patients with early-stage diseases, from whom tumor material is more widely available. Murine models helped to understand that TAN function affects not only primary tumor growth but also metastasis formation by a variety of mechanisms. In a model of acute radiation injury to the lung, locally activated neutrophils were key drivers of the tumor-supportive preconditioning of the lung TME, governed by enhanced regenerative Notch signaling, which, in turn, augmented a stemness phenotype (117). In addition, neutrophils can support lung colonization of metastasis-initiating cancer cells by producing arachidonate 5-lipoxygenase–derived leukotrienes that aid the colonization of distant tissues by selectively expanding metastasis-initiating cancer cells (118). Moreover, neutrophil extracellular traps, formed as a consequence of chronic lung inflammation caused by tobacco smoke exposure, could convert disseminated, dormant cancer cells to aggressively growing metastases (119).

## Immune cells and CSCs in the TME

CSCs represent a subset of undifferentiated cancer cells, identified in several solid tumors, with the ability of self-renewal and multilineage differentiation, responsible of tumor initiation, therapeutic resistance, and recurrence (120). Extensive efforts have been devoted to identify the molecular, phenotypic, and metabolic signatures of these cells (31–33). However, their full identification still remains challenging. Several surface antigens, including CD44, EPCAM, CD24, and CD133, have been described for CSC, but still a unique and specific combination of these markers has not been determined (121). A high metabolic activity of aldehyde dehydrogenase A1 characterizes CSCs (119). Emerging evidence has proved the influence of CSCs on immune cells, including lymphocytes, TAMs, and MDSCs, in the TME and, vice versa, the importance of immune cells in sustaining CSCs survival and stemness.

There is a limited knowledge regarding the nature and the effects of the interaction between CSCs and T- or B-cell subpopulations except for the fact that CSCs use various mechanisms to escape immunosurveillance. CSCs secrete the chemokines CCL1, CCL2, and CCL5 to recruit Treg cells (122, 123). In addition, CSCs expressed high levels of IDO1 and TGF-β, which have immunosuppressive ability and induce Treg recruitment and formation (124, 125).

Abundant data throughout multiple types of cancer support the notion that CSCs can evade CD8^+^ T-cell immunity by several mechanisms. First, they can escape cell death by downregulating MHC-I expression and thus limiting neoantigen presentation (126). Second, they can inhibit T-cell function by upregulating ligands (mainly PD-L1) of inhibitory checkpoint receptors (127). Third, they can suppress T-cell proliferation by dampening the expression of costimulatory molecules (128). To the best of our knowledge, none of these mechanisms has been reported for NSCLC so far. In NSCLC, CD8^+^ T cells are the main source of IFN-γ. Low concentrations of IFN-γ boost CSC stemness through the activation of the PI3K/AKT/NOTCH1 pathway, whereas high levels of IFN-γ induce cancer cell apoptosis through the janus kinase 1 (JAK1)/signal transducer and activator of transcription 1 (STAT1)/caspase pathway (129).

CSCs can also elude killing by NK, because NK cells can reach a functional state, known as “split anergy”, characterized by reduced cytotoxicity against CSCs and increased secretion of cytokines, including IFN-γ and TNFα, which induce CSCs differentiation (130). As an example, CSCs express reduced levels of NK-cell receptor D ligand, thus protecting them against NK lysis (131). Infiltrating myeloid cells can also interact with CSCs. Although the relative contribution of each subpopulation to cancer progression is tumor-dependent, accumulating evidence suggests that this reciprocal communication axis has a pro-tumorigenic role. CSCs–immune cell interactions have been best detailed in the context of TAMs. *In vitro* studies revealed that CSCs can have a role in monocyte recruitment across tumor types, as supernatants from patient-derived cholangiocarcinoma, hepatocellular carcinoma, or glioblastoma sphere cultures, enriched in CSCs, displayed higher levels of pro-tumoral chemokines, cytokines, growth factors, signaling molecules, and including CCL2, CCL5, CSF1, IL-13, periostin, and WISP1 if compared with their non-CSC counterparts (132–134). Among them, periostin can recruit M2 macrophages, thus augmenting tumor growth (134). In lung cancer, CSCs contribute to macrophage recruitment by secreting TNF, IL-1b, and IL-6. In the vast majority of tumor settings, including the lung, the crosstalk between CSCs and TAMs is bidirectional (135). For instance, IL-6 produced by TAMs supported the expansion and drug resistance of CSCs through STAT3 signaling in NSCLC (136). TAM recruitment to the TME, in turn, supports the CSCs’ “niche” and growth, thus enhancing the stemness of tumor cells.

## Conclusion and future perspectives

A large number of clinical trials have indicated that the use of the recently commercialized product made by autologous TILs can be considered in patients with advanced refractory solid tumors, such as metastatic NSCLC or recurrent melanoma (137, 138). Another interesting aspect of these new “immunological approaches” is the possibility to optimally cryopreserve biological material, which has opened a new chapter for transferring materials and storing these drugs over a long period. Importantly, TIL ACT can also be used in patients who are not responders to the common ICB (139). This provides substantial benefits for identifying ICB biomarkers and makes it possible to develop omics technologies, leading to an increase in computational and machine learning products (140). Consequently, physicians would be able to predict if a patient will be responsive to a defined therapy, driving the therapeutic choices and overcoming the possible resistance to TIL ACT (141).

In addition, cellular metabolism paves the way to understand the limitations of immuno- therapy and may provide rational approaches to improve anti-tumor T-cell activity by improving the metabolic fitness of anti-tumor T cells. Notably, many interventions to overcome TME inhibitory effects on T cells coincide with treatments that either directly stimulate mitochondrial activity or mimic pro-memory metabolic features (142). To tune metabolism for therapeutic interventions in cancer immunotherapy, there could be three main options: *in vitro* metabolic preconditioning, systemic *in vivo* metabolic treatments, and targeted delivery of metabolic modulators in the TME (143).

Although scientists have recently shown interest in TIL populations as a possible therapeutic strategy, other cellular components of heterogeneous solid tumors are being considered as biological targets, such as immune cells (144). In particular, the extensive involvement of TAMs in the tumor biology has captured the attention of the scientific community as it is possible to target this population through inhibition, blocking or re-education. TAMs can interact with cancer cells and CSCs, as key components of the TME (144–146). The injection of both CSCs and TAMs can increase tumor growth and the metastatic process (147, 148). Moreover, TAMs specialize in preserving the niche equilibrium and can interact with specific receptors, inducing the CSC stemness phenotype (149). However, targets involving CSCs and TAMs have not yet been defined due to the difficulty in identifying a specific CSC marker (149, 150). Contrarily, several clinical trials are investigating new targets for TAMs, aiming to inhibit their recruitment, increase their depletion, or reprogram them. Tumor immunotherapies targeting TAMs appear to be very promising, as highlighted by the recent clinical trials testing new drugs with potent anti-tumor effects (150).

Nevertheless, there are issues and limitations that need to be considered. First, TRM-derived TAMs and monocytes derived from TAMs are both found in the TME, which could be an obstacle in defining specific targets (151, 152). Second, the recruitment of monocyte-derived TAMs requires that specific receptors, such as CC2R, are inhibited, although TRMs do not appear to be recruited in this way. Third, an unspecific target of TAMs may be dangerous due to the possibility of killing the “good macrophages”, the consequences of which are yet to be determined (148, 153).

The repolarization of TAMs is also of great interest as a potential cancer therapy, although it may induce an aggressive activation of macrophages with consequent risks for patients (154, 155). To summarize, targets related to TAMs, CSCs, TILs, and immunity, in general, need to be further investigated to better define all molecular aspects that remain unclear.

## Authors contributions

BA, VM, SDB, LG, and AC wrote and approved the manuscript. SSB and LG prepared the figures. AVS, AD, MG, FS, UM, and MD read and approved the manuscript. All authors contributed to the article and approved the submitted version.

## Funding

This work is supported by Associazione Italiana per la Ricerca sul Cancro, IG grant 25073 to AC.

## Acknowledgments

SB and LG are Marylou Ingram Scholar of the International Society for Advancement of Cytometry (ISAC) for the period 2015–2020 and 2020–2025, respectively.

## Conflict of interest

The authors declare that the research was conducted in the absence of any commercial or financial relationships that could be construed as a potential conflict of interest.

## Publisher’s note

All claims expressed in this article are solely those of the authors and do not necessarily represent those of their affiliated organizations, or those of the publisher, the editors and the reviewers. Any product that may be evaluated in this article, or claim that may be made by its manufacturer, is not guaranteed or endorsed by the publisher.
